# Modification of an Airway Training Mannequin to Teach Engagement of the Hyoepiglottic Ligament

**DOI:** 10.21980/J8R06P

**Published:** 2024-04-30

**Authors:** Richard Tumminello, Daniel Patino-Calle

**Affiliations:** * Einstein Medical Center Philadelphia, Department of Emergency Medicine, Philadelphia, PA

## Abstract

**Audience:**

This airway trainer modification is designed to instruct all levels of training in emergency medicine in order to familiarize trainees with airway anatomy and obtain superior views of the glottic inlet.

**Introduction:**

During intubation with a standard geometry laryngoscope, such as the Macintosh blade, placement of the distal end of the blade within the vallecula and engagement of the median glossoepiglottic fold, also referred to as the midline vallecular fold (MVF), has long been championed by experts in airway management for its ability to improve glottic inlet visualization. This notion was further supported by the recent publication of a retrospective video review by Driver et al.^1^ Unfortunately, airway anatomy, including engagement of the MVF, does not receive the emphasis it deserves during intubation training of emergency medicine residents. Emergency physicians often have limited time to perform complete airway examinations, but a sound recognition and appreciation of the laryngeal inlet can serve as a roadmap to optimal laryngoscopy.^2^

Recent advancements in airway education emphasize visualization of airway anatomy with review of video laryngoscopy (VL) recordings to identify routine VL errors in vallecula manipulation, such as failure to engage the MVF. 3 Simulation can continue to play an essential role in enhancing trainees’ airway skills. Current airway trainers lack functional fidelity components, such an engageable MVF, resulting in a missed opportunity to teach airway skills and anatomy in a safe and controlled setting.^4^, ^5^ To address these concerns, we modified an existing airway task trainer with the addition of a simulated MVF to expose trainees to airway anatomy and adequate MVF engagement resulting in epiglottic elevation.

**Educational Objectives:**

By the end of this education session, participants should be able to:

1. Identify relevant airway anatomy during intubation, including base of the tongue, epiglottis, midline vallecular fold, anterior arytenoids.
2. Appreciate the value of a stepwise anatomically guided approach to intubation.
3. Become familiar with the midline vallecular fold and underlying anatomy, including the hyoepiglottic ligament, and how proper placement of the laryngoscope can result in improved glottic visualization.

**Educational Methods:**

The TrueCorp AirSim airway task trainer was modified with the addition of a simulated MVF. Prior to the modification described here, there were no dynamic trainers with the functional fidelity needed to teach trainees how to engage the MVF with proper placement of the distal tip of the laryngoscope. Once the trainer was created, learners are introduced to relevant anatomy through the initial lecture to unsure baseline knowledge. During the lecture, videos and images are reviewed to demonstrate the importance of an anatomical roadmap to successful intubation. Learners then practiced with the modified task trainer to gain hands-on experience with laryngoscope placement and improved glottic visualization. A short verbal debriefing was performed at the end of the skills session to address any remaining questions.

**Research Methods:**

Pre- and post-simulation surveys were completed by attendees of a weekly didactic session, ranging from medical students, PGY 1–4 emergency medicine residents, and emergency medicine attending physicians. Pre- and post-simulation familiarity with airway anatomy and comfortability with MVF engagement was assessed using a 5-point Likert scale

**Results:**

Twenty-six participants ranging from medical students to attending physicians completed a pre- and post-simulation survey. Overall, feedback from leaners was positive. Learners were excited to learn new airway management techniques and requested an expansion of current airway curriculum based on the success of this implementation.

**Discussion:**

This modified task trainer places an emphasis on teaching airway anatomy to trainees with the addition of functional fidelity by adding an engageable element providing the trainee with feedback on successful placement of the laryngoscope. This simple and cost-effective modification can add value to existing airway management curriculums by serving as a visual cue of airway anatomy and instructing trainees on proper placement of the laryngoscope. Our results showed participants experienced increased comfort with airway anatomy recognition and engagement of MVF for difficult intubations. Participants found the trainer effective with the simulated MVF resulting in glottic elevation and recommended this for future simulation. Participants were eager to learn additional airway techniques to improve their laryngoscopy skills, suggesting an area of growth for emergency medicine didactic curriculums.

**Topics:**

Airway, midline vallecular fold, anatomy, difficult airway, education.

## USER GUIDE

[Table t1-jetem-9-2-i1]

**Table t1-jetem-9-2-i1:** 

List of Resources:	
Abstract	1
User Guide	3
Instructor Materials	6

**Learner Audience:**

Medical Students, Interns, Junior Residents, Senior Residents

**Time Required for Implementation:**

This modification takes approximately 15 minutes to suture a rubber band to an existing airway trainer. After modification is complete, this modified airway trainer can be used indefinitely. Learners felt they developed recognition of airway anatomy and competence with MVF engagement following the presentation and 1–2 intubations with the modified task trainer.

**Recommended Number of Learners per Instructor:**

An instructor to learner ratio of 1:3 is sufficient when using this task trainer

**Topics:**

Airway, midline vallecular fold, anatomy, difficult airway, education.

**Objectives:**

By the end of this education session, participants should be able to:

1. Identify relevant airway anatomy during intubation, including base of the tongue, epiglottis, midline vallecular fold, anterior arytenoids.
2. Appreciate the value of a stepwise, anatomically-guided approach to intubation.
3. Become familiar with the midline vallecular fold and underlying anatomy, including the hyoepiglottic ligament, and how proper placement of the laryngoscope can result in improved glottic visualization.

### Linked objectives and methods

Learners are introduced to relevant anatomy through the initial lecture to ensure baseline understanding of knowledge (objective 1). During the presentation, video reviews of examples of adequate engagement and inadequate engagement allow the learners to gain an appreciation of what this technique can bring to their larynscopy skills as well as visualize what engagement of the MVF looks like in real-time (objectives 2 and 3). The hands-on simulation immediately following the lecture allows the learners to apply what they just learned with direct visualized feedback from the modified task trainer (objectives 2 and 3). The lecture and simulation are followed by a debriefing session which allows learners to put together everything they learned as well as a presents a final opportunity to ask questions and fill in any remaining knowledge gaps.

### Recommended pre-reading for instructor

- Driver BE, Prekker ME, Levitan RM, et al. Engagement of the median glossoepiglottic fold and laryngeal view during emergency department intubation. *Annals of Emergency Medicine.* 2021;78(6), 699–707.
- Weingart S. George Kovacs on EVLI Airway Incrementalization. EMCrit 236. EMCrit Blog. Published on October 31, 2018. Accessed on August 16th 2022. Available at https://emcrit.org/emcrit/evli/
- Trott T. The Hyoepiglottic Ligament. 5 Minute Airway. Published on November 25, 2019. Accessed on August 16^th^ 2022. Available at https://5minuteairway.com/2019/11/25/the-hyoepiglottic-ligament/

### Learner responsible content (LRC)

- Driver BE, Prekker ME, Levitan RM, et al. Engagement of the median glossoepiglottic fold and laryngeal view during emergency department intubation. *Annals of Emergency Medicine.* 2021; 78 (6), 699–707.
- Weingart S. George Kovacs on EVLI Airway Incrementalization. EMCrit 236. EMCrit Blog. Published on October 31, 2018. Accessed on August 16th 2022. Available at https://emcrit.org/emcrit/evli/
- Trott T. The Hyoepiglottic Ligament. 5 Minute Airway. Published on November 25, 2019. Accessed on August 16^th^ 2022. Available at https://5minuteairway.com/2019/11/25/the-hyoepiglottic-ligament/
- Engaging the hyoepiglottic ligament. Life in the Fast Lane. Posted February 13, 2015. At: https://intensiveblog.com/engaging-hyoepiglotticligament/

### Implementation Methods

Participants will receive a didactic presentation educating them on airway anatomy, its relevance in airway management, and video examples of adequate and inadequate MVF engagement. This didactic session is followed by a hands-on simulation in which learners use the modified task trainer. Instructors coach participants on proper MVF engagement and epiglottic elevation. The simulation portion is followed by a de-briefing session to answer questions regarding the reviewed material.

### List of items required to replicate this innovation

This innovation is designed to modify an existing airway trainer already in use in your training facility. The task trainer utilized in this modification had minor preexisting damage and had been removed from regular circulation by our simulation center, eliminating concerns about lifespan reduction of the task trainer. The additional materials will likely be at your disposal in your training center or emergency department.

1. Rubber band (https://www.staples.com/Staples-Economy-Rubber-Bands-Size-64-1-4-lb/product_143297)
2. 4-0 Suture (https://tinyurl.com/mr432a5t)
3. Suture kit (https://tinyurl.com/4p9hvx2m)

### Approximate cost of items to create this innovation

A rubber band, 4-0 suture, and suture kit was used. If not readily available, items can be purchased for approximately $20.

### Detailed methods to construct this innovation

**Figure 1:**
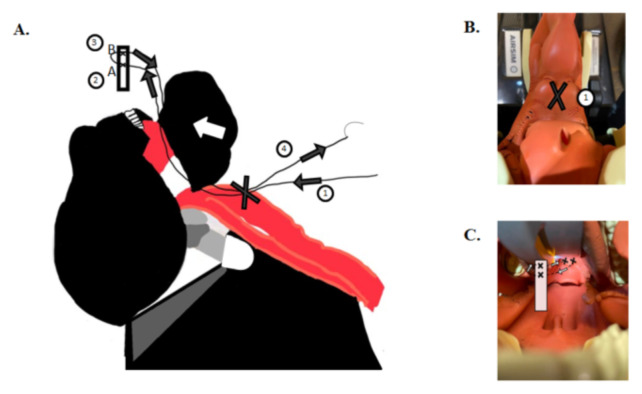
**A–C.** Author’s own image. Shows illustration correlating with Steps 1–4.

**Figure 2:**
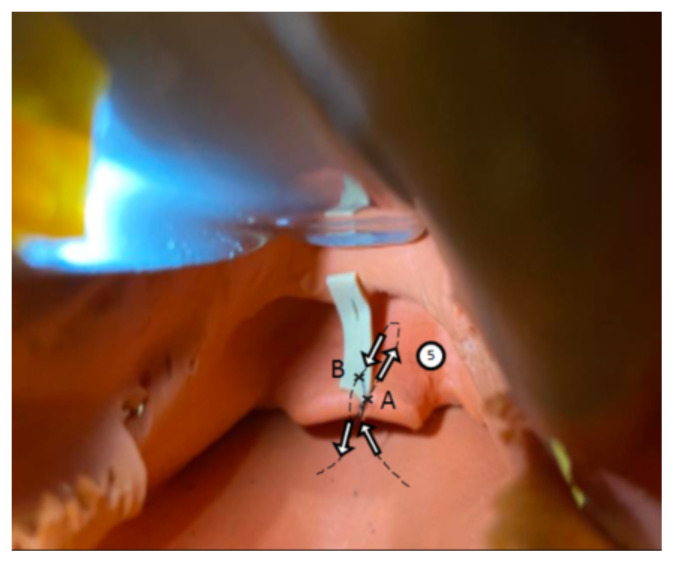
Author’s own image. Figure 2 shows photo schematic correlating with Step 5.

1. Retract the mandible superiorly revealing external surface of the larynx. Suture through outside to inside and enter the vallecular space. Retrieve needle with needle driver outside of the mouth with assistance from laryngoscope to identify the needle (Figure 1 A&B).
2. Suture through the superior end of the rubber band, cut to an approximately 2 cm segment (A), leaving enough room for a more superior re-entry point (B) (Figure 1).
3. Suture through the most superior portion of the rubber band; now bring the needle back through the mouth and exit through the back of the vallecula adjacent to initial entry point (Figure 1 A&C).
4. Holding both ends of string outside of the model, pull the rubber band down through the mouth until seated in the vallecula and secure with several knots to the outside of the larynx (Figure 1).
5. Tack down the inferior portion of the rubber band to the distal tip of the epiglottis by entering the posterior surface, driving needle through the distal portion of the rubber band (A) and returning the needle through the more superior site (B) and back through the distal epiglottis, driving the needle anteriorly to posteriorly. Secure with 3–4 simple interrupted knots (Figure 2).

### Results and tips for successful implementation

This session was implemented during an emergency medicine residency weekly didactics session. Participants completed a pre- and post-intervention assessment survey (N=26) in which comfortability was measured on a 1–5 Likert scale. Median pre-simulation and post-simulation scores were calculated with interquartile ranges (IQR). Statistical analysis was performed using a one-tailed paired T test. While our small sample size poses a significant limitation, the preliminary data presented here is promising.

Pre- and post-simulation surveys were completed by medical students, PGY 1–4 emergency medicine residents, and emergency medicine attending physicians. Pre- and post-simulation familiarity with airway anatomy and comfortability with MVF engagement was assessed using a 5-point Likert scale. In this survey, 1 represented “not comfortable at all” while 5 represented “completely comfortable.” Median pre-and post-scores were calculated with interquartile ranges (IQR).

A one-tailed paired T-test was performed for statistical significance. Satisfaction with the airway trainer to achieve the desired goal and interest in future airway lectures were also surveyed.

Twenty-six participants ranging from medical students to attending physicians completed a pre- and post-simulation survey. Three primary questions were asked of participants: 1) Comfort level with recognition of airway anatomy during laryngoscopy. 2) Comfort level with engagement of the MVF. 3) Comfort level with application and understanding of airway anatomy as a guide during a difficult intubation. Median pre-simulation scores of 4 (IQR 4.2-2), 3 (IQR 4-2), and 3 (IQR 4-2) and post-simulation scores of 5 (IQR 5-4), 5 (IQR 5-4), and 5 (IQR 5-4) were obtained, respectively. The one-tailed paired T-test showed statistical significance (p < 0.01). 95.5% of participants felt the trainer achieved the desired goal and recommended future implementation of similar sessions and advancement of airway management education.

During the simulation session, direct laryngoscopy was performed with a Macintosh 4 blade. In future sessions, we would recommend using video laryngoscopy with a standard geometry blade, such as the CMAC system, because the video component provides improved visibility of the MVF compared to direct laryngoscopy lacking video capability.

Feedback from learners was overall positive. Learners were excited to learn new airway management techniques and requested an expansion of current airway curriculum based on the success of this implementation. After 1–2 uses by the 26 participants, the rubber band remained intact without evidence of deterioration. During the learning session, the modified task trainer proved to be a cost-effective durable model. As task trainers continue to develop functional fidelity components, this modification remains a cost-effective measure to teaching hands-on skills without requiring departments to purchase new costly trainers.

The authors anticipate this modification can be transferred to other airway trainers, especially other TruCorp models which have a removable larynx cover and/or wrap-around neck covering allowing direct manipulation of the airway anatomy. The task trainer obtained for our modification was provided by our institution’s simulation center because it had previously been taken out of regular circulation due to minor damage to the simulated pharyngoepiglottic fold, making it ideal to modify.

### Associated Content

- PowerPoint Lecture

## Figures and Tables

**Figure f3-jetem-9-2-i1:**
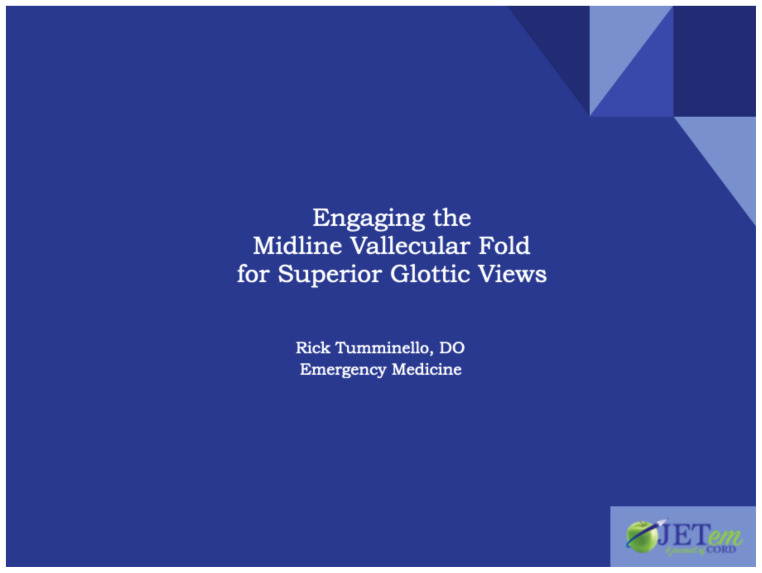
Please see associated PowerPoint file

## INSTRUCTOR MATERIALS

[Fig f3-jetem-9-2-i1]

## References

[1] Driver B, Prekker M, Levitan R (2021). Engagement of the median glossoepiglottic fold and laryngeal view during emergency department intubation. Ann Emerg Med.

[2] Kovacs G, Law A Excerpt from airway physiology and anatomy: a deep dive chapter in airway management in emergencies, the infinity edition. Airway Management in Emergencies.

[3] Barnicle R, Bracey A, Weingart S (2022). Identifying and correcting the performance errors of video laryngoscopy: The next step in emergency airway education. SAEM Pulse.

[4] Yang D, Wei YK, Xue FS (2016). Simulation-based airway management training: application and looking forward. J Anesth.

[5] Kovacs G, Levitan R, Sandeski R (2018). Clinical cadavers as a simulation resource for procedural learning. AEM Educ Train.

